# Embedding Patient and Public Engagement in a Cancer Project: Protocol for a Qualitative Study

**DOI:** 10.2196/87330

**Published:** 2026-05-11

**Authors:** Piotr Teodorowski, Marcus Arana, Daniel G Garza, Jonathan B Green, Joi Miner, Edward Duncan, Jonine Figueroa, Liz Forbat, Sarah S Jackson

**Affiliations:** 1 University of Stirling, Pathfoot Building, Stirling, FK9 4LA, United Kingdom, 44 1786 466362; 2 Community Advisory Board, Stirling, United Kingdom; 3 Division of Cancer Epidemiology and Genetics, National Cancer Institute, Bethesda, MD, United States

**Keywords:** patient involvement, public engagement, patient and public involvement, PPI, cancer

## Abstract

**Background:**

Patient and public engagement in research can enhance its quality, ensure the relevance of findings to the public, and make the process more inclusive and democratic. Ensuring meaningful engagement can be challenging and requires careful preparation.

**Objective:**

This paper presents an approach for a public engagement process in a qualitative study, describing how lived experience experts act as coresearchers from the design of the study through to analysis and dissemination.

**Methods:**

A community advisory board (CAB) comprising 4 lived experience experts will serve as coresearchers in the qualitative study. The CAB’s role spans all stages of this qualitative research. Their engagement will consist of approximately monthly meetings focusing on different research stages as the research team progresses through the study. The meetings will be designed by PT with input from the CAB to shape the discussion focus and identify relevant training needs. The lived experience experts, alongside PT, will jointly evaluate the CAB activities through regular reflective discussions and map these onto the established public engagement evaluation framework.

**Results:**

Findings from the CAB evaluation will provide insights into how meaningful the process was for both lived experience experts and the research team by capturing how lived experience experts were involved, whether their voices were heard, whether their feedback led to change, and who controlled the agenda. The study received funding in 2025, and all 4 members of the CAB were recruited in July and August 2025. In September, only the first stage was underway. As this paper reports an approach to public engagement, no findings are available at this point. The CAB evaluation is expected to take place at the end of the research project in mid-2026.

**Conclusions:**

The findings will offer a new understanding of how to engage lived experience experts, consequently providing guidance for other researchers to plan realistic engagement activities and genuinely include more members of the public with lived experience in qualitative research.

## Introduction

Patient and public engagement (also referred to as involvement) occurs when research is conducted with or by members of the public rather than to, about, or for them; thus, members of the public (eg, patients and caregivers) are actively included from the study design through to implementation and dissemination [1,2]. Members of the public engaged in research can be described in numerous ways, including lived experience experts, public contributors, or patient experts. We use “lived experience experts” as this was the preference of members of the public engaged in this project to emphasize that they bring expertise based on their previous engagement in community advisory boards (CABs) in cancer research and their lived experience of cancer. Engagement improves the quality and relevance of research findings [3] and can make health research more democratic [4]. One way for lived experience experts to be engaged is through the coresearcher role [5].

Qualitative studies have notably led to the incorporation of patient and public engagement [3,6]. Lived experience experts can assist with the development of topic guides, shaping recruitment strategies, and even data collection. Engaging them as coresearchers can lead to a more nuanced and collaborative exploration of data as they contribute unique experiences to the analysis [7,8]. However, the engagement can be challenging as some activities such as qualitative analysis can take longer than expected [8,9]. This is not necessarily a drawback, but it calls for careful preparation [10].

Descriptions of approaches for patient and public engagement activities in research are not new [11-14] but still remain limited. Detailed planning can be beneficial in qualitative research; for example, Moult et al [11] described planned activities such as data collection tool development, analysis process, and the evaluation of analysis workshops. This paper adds to this literature by detailing the approach for patient and public engagement in a qualitative study where lived experience experts will act as coresearchers included from design through to analysis and dissemination.

Some researchers might be apprehensive about involving members of the public in research [15]; others can be concerned that engagement could become tokenistic and only give an appearance of listening to public views [16]. Thus, we hope that this paper will encourage researchers to plan realistic activities and genuinely include more members of the public with lived experience in qualitative research, including analysis and dissemination.

The remainder of the paper is structured as follows. First, the research aims, background, and methods are discussed for a qualitative research project in which lived experience experts will be included as coresearchers. Second, we present the setup of the lived experience expert group, which we will refer to as the CAB. Third, we outline how the CAB will be engaged throughout the lifespan of this project and outline ethical considerations related to the engagement process. Finally, anticipated findings and a discussion are presented.

## Methods

### Research Project Supported by the CAB

This paper focuses on the CAB, and in this section, we describe the project in which they will be involved to contextualize the activities and approach we took. The study that this CAB supports aims to explore patient and public engagement in cancer research in the United States. The objectives are as follows:

1. To explore the experiences of researchers and members of the public regarding patient and public engagement in cancer research
2. To understand enablers of and barriers to engaging members of the public in cancer research
3. To capture the strategies to address engagement barriers
4. To explore the impact of patient and public engagement in cancer research

The focus will be on cancer research in the United States as this is one of the leading countries for published papers discussing patient and public engagement in health [6] and cancer research [17,18], thus offering diverse and established experiences of engaging patients and the public in cancer research. Cancer researchers have embedded patient and public engagement in their work, but recent reviews have identified some challenges and gaps in the literature [17,18]. First, members of the public have been involved in different research stages from passive to more active roles (including roles in which they shape research decisions), but most currently reported examples remain of consultative roles [17]. Engagement is reported mostly for the early parts of the research and rarely for the stages of analysis and dissemination [17,18]. Second, minoritized individuals are often underrepresented in cancer research as coresearchers [19] and in engagement activities, with studies typically involving those with higher socioeconomic status or who are already involved in patient organizations [17,18]. Third, studies do not report challenges faced in the engagement process; instead, they offer only general methodological discussions, which do not further advance engagement in cancer research [18].

### Sampling

The participants will be both researchers and members of the public. We will follow the principles of sample size in reflexive thematic analysis [20], which state that the final number of participants depends on the richness and complexity of the data collected and the ability of the data to address research objectives. Given the diversity of cancer research, we expect to conduct up to 15 interviews per participant group. This also aligns with a recent review by Hennink and Kaiser [21] of projects assessing saturation in qualitative research. Their findings suggest that qualitative interview studies, on average, require only 9 to 17 interviews.

### Inclusion and Exclusion Criteria

For researchers, participation will be open to any cancer researchers who have engaged patients or the public in research. It can be any type of cancer research (clinical, epidemiological, population science, or other). This will be limited to participants residing in the United States.

For members of the public, participation will be open to any lay members who have been engaged in cancer research in the United States. This could have been at any stage of research or level of engagement. Participants must be at least 18 years old. Those who are currently in active cancer treatment will be excluded as their participation would be an unnecessary burden.

### Interview Process

#### Overview

Interviews will be conducted remotely (online or on the phone) using semistructured topic guides. These will allow for in-depth exploration of participants’ views and perspectives. One-to-one interviews will also provide a safe space for discussion, reducing social desirability bias among participants who might not wish to share their views in front of others (such as other researchers). All interviews will be recorded, transcribed, and uploaded to NVivo (Lumivero) to facilitate the analysis.

#### Analysis

Data will be analyzed using reflexive thematic analysis by following the six steps of the analytic process as defined by Braun and Clarke [22,23]: (1) familiarization with the collected data, (2) generating initial codes, (3) searching for themes by organizing codes, (4) reviewing themes to reflect whether these work together across the codes and data, (5) defining and naming themes to ensure that each theme has a unique and clear message, and (6) writing the report for external dissemination. Everyone involved in the analysis will reflect on their positioning, background, and how these influence what they see in the data.

### CAB Members

Four lived experience experts (for clarity, we refer to them as CAB members) will be coresearchers supported by PT (lead author). CAB members will provide public perspectives and ensure that the interests of the public are represented throughout the project. Involving a group rather than a single person facilitates the engagement process [24] as it avoids generating feelings of marginalization in a lone lived experience expert when meeting the research team. The plan is for CAB members rather than researchers to attend any meetings so they can support each other. We aimed for 4 CAB members, so even if one drops out, others should be able to attend sessions. They have all been recruited from an already existing CAB focusing on cancer research as the primary requirement of lived expertise was the experience of being engaged in cancer research as coresearchers. Interested lived experience experts received an invitation email outlining the engagement opportunity. If interested, they got in touch with PT to schedule a Microsoft Teams call to discuss it further. This call did not act as a job interview but, instead, was an opportunity to learn more about the project, what the coresearcher’s role meant, and the time commitment. At the end, the lived experience experts were asked whether this opportunity was of interest to them. The places were offered on a first come, first served basis. This approach was appropriate as all potential candidates were already confident lived experience experts involved in other CABs. This also allowed for avoiding researcher bias by unintentionally choosing those who resembled the research team’s background or views. If a CAB member leaves early, the rest of the group will discuss whether to recruit a new member. Textbox 1 presents the CAB membership. Cancer diagnosis and cancer research are broad, so it was not possible to include every background. Hence, this CAB does not aim to be representative of the American population but, rather, reflects a variety of lived experiences and backgrounds.

Textbox 1. **Community advisory board (CAB) members’ biographies.**

- MA grew up in the San Joaquin Valley. Surviving as a field worker, carpenter, and house painter, MA was exposed to many pathogens. In 2012, MA was diagnosed with mucosa-associated lymphoid tissue lymphoma, which is very rare. In 2014, MA was diagnosed with neuroendocrine cancer, another rare form, which is now resolved and has no sign of recurrence or metastasis. MA has been on 2 separate cancer CABs and 2 survey CABs. In MA’s opinion, including community advisors is crucial for developing well-informed research tools and methods, which is why MA is committed to sharing their lived experience.
- JBG grew up in the mid-South and now lives in New Haven, Connecticut. With a combined background in accounting and law, he has dedicated his career to finance and administrative leadership within the higher education and nonprofit sectors. JBG was diagnosed with testicular cancer in April 2022 at the age of 47 years. Facing the diagnosis as a single person living alone, he experienced many challenges coordinating support during and after cancer treatment. JBG navigated surgery with the help of his local friends. His mother traveled to New Haven for an extended stay to support him during chemotherapy. The arrangement meant temporarily leaving his father, who has Parkinson disease, in his brother’s care. JBG has previously served on a CAB for the National Cancer Institute advising researchers working to improve the patient experience with cancer treatment and follow-up care.
- DGG is a Latino gay man living in southern California diagnosed with HIV in 2000 and anal cancer in 2015, which he survived. His lived experience with chronic illness, cancer, and mental health challenges has shaped his role as both a patient advocate and community educator. He has served on multiple CABs and remains passionate about cancer research. DGG believes that understanding the power of patient voices in guiding science, treatment, and care is crucial.
- JM is a Black woman living in Birmingham, Alabama. JM has been a member of 5 CABs (including this one), 2 that are still active, 1 that is in the study group phase, 1 that is paused indefinitely, and 1 that has not started yet. JM is a certified community health worker and has personal experience with both familial and friend cancer diagnoses. JM has provided care and support in every form, including emotional, financial, and health-centered care for her loved ones who have received these diagnoses. JM is interested in being a part of this CAB because she has seen firsthand how the voice and input of others in the community contribute to more in-depth and rich research into this and other medical issues by including the thoughts and considerations that only those with lived experience can provide.

The CAB will meet on an approximately monthly basis over a 12-month period, with some activities completed between meetings. PT will be the researcher attending and facilitating discussion at CAB meetings. We use the word “facilitation” to emphasize that the sessions will be a shared endeavor, but PT will take responsibility for any preparation to avoid burdening CAB members with the research process. CAB members will be provided with the overview of each session in advance to provide feedback that will further shape the focus and, if needed, identify their training needs. Written notes as a summary of the discussion will be shared after each meeting. If the session includes any exercises, they will be shared in advance with the CAB members to allow time for preparation and individual reflection. We present the activities planned for each step of the research process in which CAB members are involved (Table 1). Despite planning these steps, we recognize that public engagement should remain flexible to accommodate the CAB’s needs and priorities. Step 1 has already been completed, whereas the other steps are planned. This is because, without the first CAB meeting, it would not have been possible to include CAB members in the design of the engagement process.

**Table 1.** Summary of community advisory board (CAB) activities.

Step	Activities before the meeting	Meeting activities	Activities after the meeting
Induction	None	Develop ground rules for the CAB Develop topic guides for study participants Agree on the CAB role and the level of engagement	Further feedback on the draft topic guide as shared by PT Optional background reading about public engagement in research and qualitative analysis
Introduction to qualitative analysis	None	Reflection exercise Coding training	None
Data familiarization	Read 2 interview transcripts	Discussion on the 2 interviews	None
Generating initial codes	Code the 2 interviews from the previous step	Discuss completed coding and identify codes for further analysis	None
Developing and reviewing themes	None	Exploration of codes Development of initial themes and thematic map	None
Reviewing, defining, and naming themes	None	Refinement of the thematic map Development of summaries of themes	Further feedback on the summaries
Presentation of data and dissemination	Read candidate quotes	Choose relevant quotes for dissemination Discuss the GRIPP2^a^ checklist	Contribute to a paper reporting the findings
Development of lay summary	None	Draft lay summary	Further refinement of draft summary as required
Evaluation	Complete evaluation tasks (ongoing throughout the project)	Analyze experiences of working in the CAB	Contribute to the paper reporting the evaluation findings

a GRIPP2: Guidance for Reporting Involvement of Patients and the Public.

### Step 1: Induction

This first meeting took place in August 2025. This was an induction meeting that provided an overview of the research project aims, timelines, and how the CAB can be included in the study, and what the limitations of their contributions are (eg, changing the research topic or data collection method as these had already been included in the funding application). CAB members agreed on the ground rules for the CAB function.

PT presented the overview of the study aims and research methods. Thereafter, a brainstorming session was conducted to develop the interview questions for study participants (both members of the public and cancer researchers). This was followed by a discussion on questions arising from the literature developed in advance by PT. There was some overlap between the former and the latter types of questions; however, questions suggested by the members of the public demonstrated a deeper appreciation for the nuances of public engagement in cancer research than questions based on previous literature. For example, CAB members’ questions for participants aimed to capture the practicalities of everyday work in the CAB, such as handling disagreements, meeting facilitation, and personal experiences of being engaged. After the meeting, PT brought all notes together to prepare draft topic guides, which were shared with the CAB and the rest of the research team for further development.

As some CAB members expressed interest in learning more about public engagement in the United Kingdom (as PT is based at a Scottish university), some commonalities were shared during the meeting, and a postsession recommended reading included the UK Standards for Public Involvement [25]. The other learning covered the role and the importance of patient and public engagement in research. PT also recommended the chapter on qualitative analysis from a book written in lay language for lived experience experts engaged in health research [26].

### Step 2: Introduction to Qualitative Analysis

Qualitative analysis skills are often new for lived experience experts when engaged in research, but these can be one of the most notable skills acquired [27]. In this study, the CAB will be actively included in the analysis of interview data. Previous research has found that including people with lived experience can be done in a meaningful way [28] and improve the quality and rigor of the analysis, thus offering new insights that might not have been noticed by the researchers [9,29].

Data will be analyzed using reflexive thematic analysis [22,30]. This approach offers clear guidance without being prescriptive, making it an appropriate choice for novice researchers. The focus of the meeting will be to introduce the CAB members to reflexive thematic analysis. First, the 6 steps of the analysis will be outlined. Second, reflexivity and how one’s subjectivity could influence the analysis are a core component of this approach [31], so CAB members will reflect on their own backgrounds and experiences. This could overcome the challenge from a previous qualitative analysis project with lived experience experts, where researchers felt that experts blurred boundaries between analyzed data and personal experiences [10]. Depending on the outcome of this exercise, CAB members might request a more structured reflexive guide. These can help them consider reflexivity when looking at the data [9]. Third, all CAB members will code a sample of interview data from the previous project on public engagement in research during the meeting and reflect on this experience. This pilot coding should suggest whether CAB members prefer to code on paper or digitally in a Microsoft Word document using comments or whether other adjustments are needed for accessibility. It is not expected of CAB members to become independent researchers after this training but, rather, to have a generic understanding of the reflexive thematic analysis that the researchers will lead, with CAB members contributing at each stage. The research team will facilitate the analysis process by using interactive discussion methods, which are outlined in the following sections.

### Step 3: Data Familiarization

This session will take place after the initial interviews have been conducted and transcribed. Researchers will conduct interviews, and a professional transcription company will transcribe them. These transcriptions will be shared with CAB members in advance so they can familiarize themselves with the data and take notes about similarities and differences between participants. They will also be able to identify where the interviewer could have asked follow-up questions or explored different avenues of inquiry. This will become a reflective space to shape future data collection. The experience of familiarity with the first interviews will allow CAB members to determine how much time commitment the coding process might entail in the later stages. It will be their decision how many interviews they wish to read or code depending on their time availability. Previous experiences have shown that coding even one interview by lived experience experts can improve the quality of data analysis and offer new insights into findings [9,29].

### Step 4: Generating Initial Codes

CAB members and PT will code the assigned interviews and bring them to the session. Each person will be asked to share their observations on the data and codes and, similarly to Cotterell [8], take note of interesting data segments. This inductive approach will help identify relevant codes for the remaining interviews. PT will then code the rest of the dataset in NVivo (version 20) with input from another researcher who will act as a “critical friend,” reviewing coding and providing feedback [32]. Coding is an iterative process, so it is expected that the initial codes will evolve throughout this process, and if significant changes are made, these will be brought back for discussion with the CAB. The reason why the CAB will not be involved in coding all interviews is that, when informed about the time commitment for this step of qualitative analysis, they reflected that it was too time-consuming and were happy for PT to lead it.

### Step 5: Developing and Reviewing Themes

As all data will be coded, PT will bring the organized data to generate themes. It is expected that the discussion will be led by a researcher but with contributions from CAB members who will actively shape the product at both the initial and final stages of theme development [9]. PT will present the content captured in each code, and the CAB members will participate in a brainstorming activity to organize the codes into initial themes. We will develop an initial thematic map through this exercise, with PT developing it throughout the discussion. Thematic maps are a popular way in qualitative analysis to capture connections among themes, subthemes, and codes visually [33,34]. They have been successfully used during focus group discussions to keep the discussion on track, ensure transparency, and empower participants [35].

Depending on the number of codes, more than one session focusing on searching for themes might take place. It is not uncommon at this stage of the analysis to recognize that it is necessary to take a step back and recode some of the data as the codes might be revised [33]. If this happens, it will be a joint decision between the research team and CAB members.

### Step 6: Reviewing, Defining, and Naming Themes

This session will start with the review of candidate themes developed previously. Using the previous discussion, PT will update the draft thematic map exhibiting the themes and their connections. If the CAB agrees on the representation, we will move on to defining and naming themes. We will assign themes to each person, who will then write a short summary (or bullet points) of that theme (including subthemes) to share with the group for further joint iterations.

### Step 7: Presentation of Data and Dissemination

The final step of reflexive thematic analysis involves writing up a paper reporting on the findings. Lived experience experts can contribute as coauthors of academic papers [36]. In this project, CAB members will first contribute to the selection of quotes used in the paper to illustrate each theme. Similarly to Hemming et al [9], we will bring relevant quotes to the meeting, but rather than ranking them, the CAB will try to establish a consensus through discussion. PT will take the lead on writing the paper and share it for feedback and edits with the rest of the research team and CAB, who will be invited to be coauthors. We will complete the Guidance for Reporting Involvement of Patients and the Public (GRIPP2) checklist [37] to ensure appropriate reporting of the public engagement process in the findings paper.

### Step 8: Development of Lay Summary

After the submission of the findings paper, the CAB will reconvene to prepare a lay summary that will be shared with participants who requested it and the public on social media.

### Step 9: Evaluation

Understanding what works for whom and under what circumstances has been recognized as an essential element in patient and public engagement [38]. However, it is not always well documented or evaluated [39]. There are some papers reporting on the evaluation of public engagement in cancer research [40-43]. More evidence is needed as patient and public engagement activities in cancer research are not often evaluated [17,18].

The CAB decided to coevaluate their engagement in the research project as an ongoing process. Both the CAB members and PT will complete data collection for the evaluation and jointly analyze the findings. This will help ensure that, where improvements are possible, these can be incorporated into the sessions by the research team. After every 3 meetings, the CAB members and PT will answer three reflective questions: (1) what worked well? (2) What did not work well? (3) What did surprise you?

These can be completed in writing or through a recorded video of no longer than 3 minutes. These experiences will be shared at the start of the following meeting for a joint reflection.

At the end of the project, we will map our experiences on the cube evaluation framework [44,45]. The framework allows for the capture of experiences on a scale (high vs low), with the CAB members and PT each placing their answer on the scale for one of four issues: (1) whether CAB members had multiple opportunities and approaches to shape the project, (2) who controlled the focus of the sessions, (3) whether CAB members felt that their voices were heard, and (4) whether they felt that their contributions were implemented. Each individual’s score will be mapped to the scale in 0.5-point increments. If more than one person records their answer in the same location, the bullet point size will increase by 0.5 points for each person. This will allow us to capture where there were agreements. The findings from this mapping exercise will be presented as a diagram that merges all scales. Clusters of answers around the center of the diagram will suggest poorer-quality engagement, and those toward the extremes will represent stronger, higher-quality engagement. This will allow us to compare scores across all 4 issues, identifying whether one worked well but others did not. To illustrate the mapping decision, the group will use their postmeeting reflections to provide a narrative description. These will be collated into a table to summarize their individual experiences and then jointly synthesized into narrative common themes. Jointly evaluating the process will create a collaborative space for reflection among CAB members and PT and help reflect on the engagement process from a long-term perspective.

### Ethical Considerations

Usually, ethics approval is not required for patient and public engagement in research [46,47]. However, as CAB members will act as coresearchers and have access to the interview transcripts, this paper was a part of the ethics application to provide details on how lived experience experts will contribute to the data analysis process, with CAB members included as ethics coapplicants. Ethics approval has been granted by the University of Stirling General University Ethics Panel (number 23682). We also recognize that there are ethical principles regarding patient and public engagement in research [46] and considered them when designing the CAB’s activities.

First, a relationship between researchers and CAB members underpins the productive public engagement process. CAB members, along with PT, agreed on the ground rules for sessions, which incorporated principles of being in a safe space and not sharing private experiences outside of the group unless approved by the individual whose experience will be shared. CAB members also have the option to participate in any activity they wish to, and debriefing opportunities are available after meetings directly with PT or through peer discussion on WhatsApp. Online discussions through a Facebook group [48] and a WhatsApp chat [40] have been used previously in public engagement. We decided to use WhatsApp as it is free, does not require a private account, and can also facilitate one-to-one calls or conversations between international numbers (which was relevant as CAB members are US based whereas PT is in the United Kingdom). If it would be relevant for another researcher to join the meeting, this will first be discussed with the CAB. PT will let CAB members know at least a week in advance and explain why it would be relevant for that researcher to join the next meeting.

Second, public engagement must ensure power sharing between researchers and the CAB. There is a risk of a power imbalance, so it is relevant to ensure that public voices are respected and have equal value to researchers. CAB members have been engaged from the study design stage to ensure that they can influence the research as early as possible. The CAB members agreed on the terms of reference that recognize their responsibilities but also those of the research team. CAB members commit to provide a public perspective on all research aspects; attend meetings; give constructive input and contribute actively to discussions (which might include reading information between the meetings); maintain confidentiality on all project-related information; and, if invited to be a part of the dissemination, contribute to writing and editing. Ensuring the inclusion of a public perspective helps researchers deliver more relevant research but also gives space for CAB members to present issues that are important to them [49]. On the other hand, researchers are expected to listen to and implement feedback from CAB members where appropriate. If it is not possible to do it, they will explain the reasoning and discuss it with the CAB. Explaining how feedback has been used is important so that CAB members know how their contributions impact the research [43]. CAB members will also be credited in any publication for their contribution; where appropriate, this will include coauthorship. Researchers will provide appropriate support and training to CAB members to ensure that they are adequately equipped to fulfill their role [50]. All documentation will be shared first via email and then organized into folders on Microsoft SharePoint, accessible by everyone. CAB members will receive regular updates on the progress, and their contributions will be acknowledged. These progress updates will be provided via email and at the start of each meeting as a progress summary. When the CAB members coauthor any dissemination, they will receive emails at each stage of the review process. The terms of reference also specify the reimbursement level, which has been settled at US $100 per meeting.

The ongoing evaluation process will ensure that all ethical considerations are appropriately addressed from the perspectives of CAB members and researchers. If there are areas for improvement, for example, in providing training, the research team will address them at the next meeting.

## Results

The study received funding in 2025, and all 4 members of the CAB were recruited in July and August 2025. In September, only the first stage was underway. As this paper reports an approach to public engagement, no findings are available at this point. The CAB evaluation is expected to take place at the end of the research project in mid-2026 with results to be published in October 2026. Data collection will be ongoing throughout the whole project.

## Discussion

### Anticipated Results

The results from the evaluation will provide insights into how meaningful the process was. First, the results will explore whether CAB members and PT felt that public voices made a difference and how they affected the overall project. Second, they will explore whether CAB members had a broad range of opportunities to be involved in the research or whether these were limited. Third, they will explore whether the overall project was more focused on what the research team saw as a priority or on what the CAB saw as a priority. Fourth, they will capture how and whether the research team incorporated the CAB’s contributions.

### Comparison to Prior Work

Public engagement in qualitative data analysis can offer new opportunities for lived experience experts to connect with similar-minded people who also share lived experiences and help some of them find a purpose, thus potentially improving their emotional well-being [27]. Working online has drawbacks, but the evidence from moving public engagement online has shown that it is possible to ensure that it remains meaningful for lived experience experts [51]. However, how online meetings look should be codeveloped with lived experience experts to ensure accessibility and that everyone can contribute [52], which we undertook during the development of this approach to public engagement.

Engaging lived experience experts as coresearchers during the qualitative analysis process can benefit both sides [27] as researchers understand public needs better, whereas the lived experience experts gain academic skills [10] alongside project planning, meaning making, and dissemination that can be transferable to other opportunities. Thus, both groups learn from each other and appreciate differences and what can be achieved when coming together [8]. This will be particularly relevant in this research project as it aims to better understand patient and public engagement in cancer research. Public engagement in research can support the empowerment of lived experience experts [53]. Actively engaging lived experience experts has the potential to reduce power imbalances in research, not only in this project but also in other cancer research studies that might incorporate our findings.

### Challenges and Limitations

Public engagement in research has been described as a “ladder,” with the top step representing the most power given to lived experience experts and the bottom step representing having limited (or tokenistic) influence on the research [54-56]. Our project aims to be as close to the top as possible by actively sharing power between researchers and CAB members. However, we recognize that some activities might be more consultative than shared leadership. For example, the overall research project and its aims have been previously defined in collaboration with an external funder, but CAB members still have an opportunity to define what questions participants will be asked, shape the analysis, and decide on the presentation of the findings. Some activities will be led by researchers (such as coding a dataset or writing academic papers), whereas others (shaping themes and writing lay summaries) will be led by the CAB.

### Future Directions and Dissemination

There is an increase in literature describing the plans for public engagement in research [11-14] and evaluation of advisory groups and boards [40,57,58]. We plan to contribute to this growing literature through this paper and later publish the findings of our CAB evaluation.

### Conclusions

This paper outlines a thoroughly planned engagement approach for a qualitative research project with the contribution of CAB members. One of the strengths of this protocol is its flexibility and adjustability as CAB engagement stages can be tailored (eg, by adjusting the length of the discussion) based on ongoing evaluation. This has the potential to greatly impact the research project by increasing its quality, as well as benefiting the research team and the CAB.

## Abbreviations

CAB: community advisory board
GRIPP2: Guidance for Reporting Involvement of Patients and the Public
